# Supplementary material for: Patterns of Intron Gain and Loss in Fungi
Source: PLoS Biol. 2004 Nov 30;2(12):e422. doi: 10.1371/journal.pbio.0020422 (PMC532390; doi:10.1371/journal.pbio.0020422)
Supplement: Table S1 — Also available at http://genes.mit.edu/NielsenEtAl/. (4.3 MB ZIP). [file pbio.0020422.st001.zip › NielsenEtAl/html/674.html]

AN4940.1.NCU04415.1.MG05164.1.FG11602.1


```
 CLUSTAL W (1.82) Multiple Sequence Alignments - Introns Inserted


Sequence 1: NCU04415.1	561 aa
Sequence 2: MG05164.1	542 aa
Sequence 3: FG11602.1	548 aa
Sequence 4: AN4940.1	533 aa
Alignment Length: 571 aa
Number Identitical Residues: 207 aa
Alignment Score (without introns) 11891


MG05164.1 	--MRVLTTLVNLALVAGAV-GSNWF--TKAA1YNKWHETELERWLSDHDVPYPTPADRRD
NCU04415.1	--MRIAKTFLSLALVADAVYGSSWF--SNAA1YNKWHETELERWLSDHDVPYPTPADRKE
FG11602.1 	--MRFVNAIV-AAVAINSVAASTWFPGSKPV1YNKWHETELERWLSDHDVPYPTPADRKD
AN4940.1  	MKLQLASWLVCALATAEAVGASNWF--SKAV1YNKWHETELERWLADHGTDTSSSFYCRK
          	 .::. . ::    .  :* .*.**  ::.. *************:**..  .:.   :.

MG05164.1 	LEKLVQQNWDSYVVQPYSKWDNQQLADYLKLKGVETKDSAQASRDSLVSQVQKSWYETED
NCU04415.1	LEKLVQSNWDNQVVAPYKEWDTAKLTDYLKQKGIETKDSAGSTKDSLIDQVKGVWYESED
FG11602.1 	LESLIEKNWDAYVVSPYNSWDTNQLSAYLQSKGKETKNEAAASKDSLLSQVKANWYETEN
AN4940.1  	LT----------VVS-----------DIPYPSPADRKELENAVKTNWDLKVQKPLGQLSD
          	*           **                 .  : *:   : : .   :*:    : .:

MG05164.1 	KAQQSYSSVKDWILDT~WTESQLKSFCDHNNIPV~PQPRQRDTILTKARESYDTVAQKFG
NCU04415.1	KAHNSWLNVKDWILDS~WTDSQLKAFCDRHSIPV1PQPRKRDTLLEKARSSYETIAQKAG
FG11602.1 	NAHSAWLNVKDWILDT~WTESQLKAFADKHGIPA1PQPRQRDTLLQKARSSYEAIAKKAN
AN4940.1  	HSGDRWEHTKEWIFDT2WTDSQLKAFLDRHGIPS~PQPRRRDTLLKAARENYEAVAKKLG
          	:: . :  .*:**:*: **:****:* *::.**  ****:***:*  **..*:::*:* .

MG05164.1 	EAVAYPGNWLYETWSESDLKEWLDTHGFPAPQPSS~RDKLIASVRRNSRLAYLKLQADSA
NCU04415.1	ETAAYPGNWLYETWSESDLKEWLDTHGIPAPQPTT0RDKLIATVRRNSRLAYLRMKEQQA
FG11602.1 	QASSYPGNWLYETWSESDLKEWLDTHGFPAPQPMT~RDKLIASVRRNSRLAYLKAQDQAA
AN4940.1  	EAAHYPGNWLYDQWSDSDLKLWLDERGWPAPQPTT~RDRLIAAVRRNTRLASLQAKNIAA
          	::  *******: **:**** *** :* ***** : **:***:****:*** *: :   *

MG05164.1 	SASKSAQDAYATVTDKLIDAWSESDLKDFADRNGIPV1PQGTKVNELRSLVRKHRADILG
NCU04415.1	SATSSAQAAYATLTDKLIDAWSESQLKDFCDKNGIPV~PQGTKLNQLRALVRKHRADLLG
FG11602.1 	SATASAQGAYATLTDMIIDAWSESQLKEFCDKNGIAV~PQGTKANELRALVRKNRADILG
AN4940.1  	SVVQSANAAQETLSEALFSAWSDSDLKRFLDEHGIHV~PQGSKRNELIALARKHRAALVD
          	*.  **: *  *::: ::.***:*:** * *.:** * ***:* *:* :*.**:** ::.

MG05164.1 	DNISGS------AASAYGAATSKAGNTYASATDSASLAAQEAFDKSVNTWSESRLKAYLD
NCU04415.1	DTLSAS------ASSAFGAATSNIGSNYAKATDAASQAAADAFDKAINTWSETRLKAYLD
FG11602.1 	DNLSAS------AASAFGAATSNAQNQYAKATDSASLAAQDAFNQALGTWSDTRLKSYLD
AN4940.1  	QASSASSSVSSTASEYIGAATSRAGNEYARATDNASLKSQEAFDTAINSWSDSRLKAFLD
          	:  *.*:: :::*:.  *****.  . ** *** **  : :**: ::.:**::***::**

MG05164.1 	ARGVPVPQTSKVDELRALVRKHSHKAATQWSSWNFEDYSIENLK~NYLMANGDAAAKKAA
NCU04415.1	ARGVPVPQASKIDELRALARKHAHKAASGYTAWTFDDWTYDNLK~DYILAHGDAAGKKVA
FG11602.1 	ARGVPVPQGSKKNELEALVRKNAHVAAHGGNAWTFDDFSYDNLK~KYLERNGDAAAKQIA
AN4940.1  	ARGVPVPQKNKRDELLAQVRLNKHKAATGWSAWTFDTWDTEHLK2KYLS----SVNSKVA
          	******** .* :** * .* : * **   .:*.*: :  ::** .*:     :. .: *

MG05164.1 	AKTDATRADLVKAAQSAYGSASKAGGDTYASATSYLAQATQAAKDTTFETWTDSELKAYL
NCU04415.1	NQAGVTRDQLLAAAQSGYASASAAGGDSYASVTSYLAKATDSAKAQTFDTWSESDLKAYL
FG11602.1 	EKKDATRDELYDAAQSAYSSASSAGGSTWASATSYLTSATNSAKQSAFDEWTETDLKAYL
AN4940.1  	HRAGVTRDELVKEAQDTYEKATKAGGSQLASATSYLKQATASAKDATFDTWSDSDLKQYL
          	 : ..** :*   **. * .*: ***.  **.**** .** :**  :*: *::::** **

MG05164.1 	DSYGV0PVPQGSKTEELRAWARKQWMYFQYGTTTPAGTMFAKLTEGFKGGVEWIKGQLNM
NCU04415.1	DSYGV0PAPQGSTLNELRALARRQWTYFKYGTSTPTETVFAKIGESAKAGWDWIKGQLHI
FG11602.1 	DSYGV~SVPQGSKLEELKAQARKQATYFKYGTSSPSGTLFAKLGDTARDTYNWVASQLQL
AN4940.1  	DSYGV~PVYQGTELNELRAAARRNAHWFRYGTSTPQGALYAKFTGAAQWLLDQLKIGAAS
          	***** .. **:  :**:* **::  :*:***::*  :::**:    :   : :      

MG05164.1 	GADAAKKAADDANAKAKEEL------------------
NCU04415.1	GADAAQKQAIKAGDKVKEGADSYQKGAEKVGDKIKEEL
FG11602.1 	GGQAAKEKAAQVEAEAEAKVR--------------EEL
AN4940.1  	GRAQGREAAEKLQDKAEKKAEEVR-----------SEL
          	*   .:: * .   :.:    .             ..
```
